# Application of γ-aminobutyric acid alleviates salinity-mediated growth decline and oxidative damage by increasing proline and antioxidant functioning in olive (*Olea europaea* L.)

**DOI:** 10.3389/fpls.2025.1737858

**Published:** 2026-01-13

**Authors:** Taghreed S. Alnusaire

**Affiliations:** Department of Biology, College of Science, Jouf University, Sakaka, Saudi Arabia

**Keywords:** antioxidant enzymes, ion homeostasis, nutrient uptake, osmolyte accumulation, oxidative stress, salinity stress, γ-aminobutyric acid

## Abstract

Salinity stress is a major environmental constraint limiting crop productivity by inducing osmotic imbalance, ion toxicity, and oxidative damage. This study investigated the potential of exogenous γ-aminobutyric acid (GABA) to enhance plant tolerance under saline conditions. Plants were subjected to three salinity levels (0, 100, 150 mM NaCl) with or without foliar application of GABA (0, 1, 2 mM). Salinity significantly reduced growth parameters, including plant height, leaf area, and biomass accumulation, while increasing oxidative stress markers such as malondialdehyde (MDA), hydrogen peroxide (H_2_O_2_), and superoxide anions (O_2_•^−^). However, GABA application, particularly at 1 mM, substantially mitigated these negative effects. GABA-treated plants exhibited higher relative water content (RWC), membrane stability index (MSI), and accumulation of osmolytes (proline, soluble sugars), reflecting improved water status and metabolic adjustment. Antioxidant defense was enhanced, with increased activities of superoxide dismutase (SOD), catalase (CAT), peroxidase (POD), ascorbate peroxidase (APX), and glutathione reductase (GR), alongside elevated levels of ascorbate (AsA), glutathione (GSH), and favorable redox ratios (AsA/DHA, GSH/GSSG). GABA also improved nutrient uptake by increasing macronutrient (N, P, K, Ca) and micronutrient (Fe, Mn, Zn, Cu) levels while reducing Na^+^ accumulation and Na^+^/K^+^ ratios. Multivariate analyses (heatmap, PCA) revealed that GABA-treated plants under moderate salinity closely clustered with non-stressed controls, highlighting its protective role. Overall, GABA enhances salinity tolerance by modulating osmotic balance, ion homeostasis, and antioxidant defense mechanisms, supporting its use as a promising agrochemical for improving plant resilience under salt stress.

## 1 Introduction

Olive (*Olea europaea* L.), a major perennial fruit tree of arid and semi-arid regions, is often exposed to high soil salinity, especially in the Mediterranean basin where climate change and irrigation with brackish water exacerbate soil salinization (Boussadia et al., 2023; El Yamani and Cordovilla, 2024). Salinity stress is a critical abiotic constraint that significantly limits plant growth and agricultural productivity worldwide (Weisany et al., 2012). Excess salt in the root zone imposes both osmotic stress (water deficit) and ionic toxicity (Na^+^ and Cl^–^ accumulation) on olive plants, disrupting water uptake, nutrient balance, and various physiological processes (Weisany et al., 2024). Consequently, salt-stressed olive trees exhibit stunted growth, leaf chlorosis or necrosis, and pronounced declines in gas exchange and photosynthetic capacity, ultimately reducing yield (Regni et al., 2019). Although olive is considered a moderately salt-tolerant species, significant genotypic differences exist in salinity tolerance – even relatively mild salinity can adversely affect sensitive cultivars (Boussadia et al., 2023; El Yamani and Cordovilla, 2024). This variability underlines the importance of understanding olive’s stress physiology and developing strategies to mitigate salinity-induced growth decline in this crop.

γ-Aminobutyric acid (GABA) is a four-carbon non-protein amino acid that accumulates rapidly in plants under salt stress and functions as an important stress signal and metabolite (Jalil and Ansari, 2020). Exogenous GABA applications have shown broad effectiveness in alleviating salt stress in many plant species. By supplying GABA externally (via foliar spray or nutrient solution), researchers observe mitigation of both the osmotic and oxidative components of salinity stress (Li et al., 2022). GABA-treated plants maintain higher tissue water content and turgor under salt stress, partly by accumulating more osmoprotectants such as proline and soluble sugars that help retain water. (Dabravolski and Isayenkov, 2023) Another key mechanism by which GABA confers salt tolerance is by enhancing antioxidant defenses. GABA signaling boosts the activities of major antioxidant enzymes and upregulates their genes under stress (Li et al., 2022). GABA-treated plants typically show higher activity of SOD, CAT, peroxidases (ascorbate peroxidase), and other ROS-scavenging enzymes than untreated plants under the same salt stress (Li et al., 2022).

Salt stress imposes oxidative stress on plants by overproducing ROS (such as O_2_^−^•, H_2_O_2_, OH•) in chloroplasts, mitochondria, peroxisomes, due to ion toxicity and osmotic imbalance (Al Kharusi et al., 2019). To prevent ROS-induced damage (lipid peroxidation, protein oxidation, DNA damage), plants rely on a robust antioxidant defense system (Ahmad et al., 2019). Consequently, enhancing antioxidant defense (through genetic selection, metabolic priming, or exogenous protectants like GABA, proline, glycine betaine) is a recognized approach to improve crop performance on saline soils. By combining osmolyte accumulation (proline, GABA) with a fortified antioxidant network, plants can better maintain cellular homeostasis and productivity under salt stress (Hasanuzzaman et al., 2014).

Effective salinity tolerance in plants like olive involves a combination of mechanisms: osmotic adjustment (through proline and other osmolytes), ion homeostasis, and a robust antioxidant system to mitigate oxidative injury. Exogenous GABA application intriguingly ties into these processes by inducing osmolyte accumulation (notably proline) and upregulating antioxidant defenses, thus directly addressing two major facets of salt stress injury. However, most research on GABA-mediated salinity alleviation has focused on annual model or crop species, whereas studies in long-lived woody fruit crops like olive remain limited (Al-Khayri et al., 2024). The extent to which exogenous GABA can improve olive salt tolerance – particularly via modulation of proline metabolism and antioxidant enzymes is not yet fully understood. Therefore, the present study was undertaken to evaluate whether exogenous GABA application can alleviate salinity-mediated growth decline and oxidative damage in olive by enhancing proline accumulation and antioxidant functioning. By elucidating GABA’s influence on these physiological and biochemical responses, this work aims to advance our understanding of stress mitigation in olives and to inform the development of GABA-based strategies for improving salt tolerance in woody crop species.

## 2 Materials and methods

### 2.1 Plant material and growth conditions

Uniform, healthy olive (*Olea europaea* L.) seedlings of the cultivar ‘Arbequina’ were obtained from the Bosita area in 2025, Al Jouf, Saudi Arabia. Seedlings were approximately four months old and had 6–8 fully expanded leaves. They were transplanted into 3 L plastic pots containing a homogenized growth medium composed of sandy loam soil, peat moss, and perlite in a 2:1:1 (v/v/v) ratio. The pots were maintained in a controlled greenhouse at 25 ± 2 °C during the day, 18 ± 2 °C at night, 60–70% relative humidity, and a 14/10 h (light/dark) photoperiod with a photosynthetic photon flux density of 350–400 µmol m^−^² s^−1^. Plants were irrigated with tap water to 70% field capacity three times per week and fertilized weekly with one-tenth-strength Hoagland nutrient solution to ensure uniform establishment prior to treatments.

### 2.2 Experimental design and treatments

After a three-week acclimatization period, the plants were subjected to a factorial experiment (3 × 3) arranged in a randomized complete block design (RCBD) with three salinity levels and three concentrations of γ-aminobutyric acid (GABA). Each treatment was replicated four times, and each replicate consisted of three plants. This experimental layout was designed to evaluate the interactive effects of salinity stress and exogenous GABA application on plant physiological and biochemical responses.

Salinity treatments were imposed using sodium chloride (NaCl) solutions at three concentrations: 0 mM NaCl (no salinity; control), 100 mM NaCl (moderate stress), and 150 mM NaCl (severe stress). These levels were selected to represent a gradient from non-saline to high salinity conditions, enabling assessment of plant responses under increasing osmotic and ionic stress. Foliar GABA treatments were applied at three concentrations: 0 mM (no GABA; control treatment with distilled water + 0.05% Tween-20 as surfactant), 1 mM GABA, and 2 mM GABA. The selected GABA concentrations were based on previous reports indicating their effectiveness in alleviating salinity-induced oxidative and ionic stress without phytotoxic effects (Ullah et al., 2023; Wang et al., 2023). Specifically, concentrations of 1–2 mM GABA have been shown to enhance antioxidant enzyme activities, osmolyte accumulation, and ion homeostasis in various crops under salinity and drought stress. The full factorial design (3 salinity levels × 3 GABA concentrations) yielded nine treatment combinations, as outlined in **Table 1**.

**Table 1:** Physicochemical properties of the experimental soil before treatment application.

Sand (%)	Silt (%)	Clay (%)	Texture Class	pH	EC (dS m^−1^)	OC (%)	N (%)	P (mg kg^−1^)	K (mg kg^−1^)	Fe (µg g^−1^ DW)	Mn (µg g^−1^ DW)	Zn (µg g^−1^ DW)	Cu (µg g^−1^ DW)
41.5	31.2	27.3	Loam	7.18	0.82	0.97	0.82	106.4	495.8	198.6	122.5	72.8	24.1

EC, Electrical Conductivity; OC, Organic Carbon; N, Nitrogen; P, Phosphorus; K, Potassium; Fe, Iron; Mn, Manganese; Zn, Zinc; Cu, Copper; pH, potential of Hydrogen.

Salinity stress was induced by irrigating the plants with NaCl solutions prepared in distilled water. To avoid osmotic shock, the NaCl concentration was gradually increased to the target levels in three steps (⅓, ⅔, and full strength over three consecutive days). The soil electrical conductivity (ECe) was monitored weekly using a portable conductivity meter to ensure stable target salinity levels of approximately 1.2 dS m^−1^ (S_0_), 10.5 dS m^−1^ (S_1_), and 15.8 dS m^−1^ (S_2_) throughout the experimental period.

Foliar GABA treatments were applied using a hand sprayer, ensuring uniform coverage until complete leaf wetness (approximately 40 mL solution per plant). The first GABA spray was applied two days before salinity initiation to serve as a priming treatment, followed by weekly foliar applications for six consecutive weeks. All sprays were performed in the morning (8:00–10:00 a.m.) under calm wind conditions to minimize drift and ensure consistent absorption. This experimental setup was intended to investigate how exogenous GABA supplementation mitigates the detrimental effects of salinity on plant growth and stress physiology.

### 2.3 Growth and biomass determination

After six weeks of treatment, plants were harvested and separated into shoots and roots. Plant height, leaf number, and leaf area (using a LI-3100C leaf area meter, LI-COR, USA) were recorded. Fresh weights of shoots and roots were measured immediately, and dry weights were obtained after oven-drying at 70 °C for 72 h until constant weight.

### 2.4 Relative water content and membrane stability index

RWC was determined following Weatherley (1950). Fresh leaves were weighed (FW), floated in distilled water for 6 h to obtain turgid weight (TW), and then oven-dried at 70 °C to constant weight (DW).


$$\text{RWC}\left(\%\right)=\frac{FW-DW}{TW-DW}\times 100$$

MSI was measured according to Sairam et al. (1997). Leaf discs (0.2 g) were immersed in two sets of test tubes containing 10 mL of double-distilled water. One set was heated at 40 °C for 30 min (C_1_), and the second set at 100 °C for 10 min (C_2_).


$$\text{MSI}\left(\%\right)=\left(1-\frac{C_{1}}{C_{2}}\right)\times 100$$

### 2.5 Determination of ionic content

For Na^+^ and K^+^ determination, oven-dried leaf and root samples were digested in 1 N HNO_3_. Ion concentrations were quantified using a flame photometer (Model 410, Sherwood Scientific Ltd., UK), and the Na^+^/K^+^ ratio was calculated.

### 2.6 Proline

Proline content in leaf tissues was determined following the acid-ninhydrin method described by Bates et al. (1973). Approximately 0.5 g of fresh leaf tissue was homogenized in 10 mL of 3% (w/v) sulfosalicylic acid and filtered through Whatman No. 2 filter paper. Two milliliters of the filtrate were reacted with 2 mL of acid-ninhydrin solution (1.25 g ninhydrin dissolved in 30 mL glacial acetic acid and 20 mL of 6 M phosphoric acid) and 2 mL of glacial acetic acid in a test tube. The mixture was heated at 100 °C for 1 hour in a water bath, and the reaction was terminated by placing the tubes in an ice bath. The chromophore was extracted with 4 mL of toluene, and the absorbance of the upper phase was measured at 520 nm using a UV–visible spectrophotometer (Shimadzu UV-1800, Japan). Proline concentration was calculated using a standard curve prepared with known concentrations of L-proline and expressed as µmol proline per gram fresh weight (µmol g^−1^ FW).

### 2.7 Soluble sugar and protein

Soluble sugars were quantified using the anthrone method. Fresh plant tissue (approximately 0.2 g) was homogenized in 5 mL of 80% ethanol and incubated in a water bath at 80 °C for 30 minutes. The extract was centrifuged at 5000 rpm for 10 minutes, and the supernatant was collected. To 1 mL of extract, 5 mL of freshly prepared anthrone reagent (150 mg anthrone dissolved in 100 mL of concentrated sulfuric acid) was added (Irigoyen et al., 1992). The mixture was heated in a boiling water bath for 10 minutes, cooled rapidly in an ice bath, and the absorbance was measured at 620 nm using a UV–visible spectrophotometer (Shimadzu UV-1800, Japan). Glucose was used as the standard, and results were expressed as mg soluble sugars per gram fresh weight (mg g^−1^ FW). and soluble protein content was measured following Bradford (1976) using bovine serum albumin as standard.

### 2.8 Lipid peroxidation

Lipid peroxidation in plant tissues was assessed by quantifying malondialdehyde (MDA) levels, a byproduct of polyunsaturated fatty acid peroxidation, using the thiobarbituric acid reactive substances (TBARS) method described by Heath and Packer (1968). Fresh leaf samples (0.5 g) were homogenized in 5 mL of 0.1% (w/v) trichloroacetic acid (TCA) using a chilled mortar and pestle. The homogenate was centrifuged at 10,000 × g for 10 minutes at 4 °C. To 1 mL of the supernatant, 4 mL of 0.5% (w/v) thiobarbituric acid (TBA) prepared in 20% TCA was added. The mixture was incubated in a water bath at 95 °C for 30 minutes, then quickly cooled in an ice bath to stop the reaction. Samples were centrifuged again at 10,000 × g for 5 minutes, and the absorbance of the supernatant was measured at 532 nm and corrected for non-specific turbidity by subtracting absorbance at 600 nm. MDA concentration was calculated using an extinction coefficient of 155 mM^−1^ cm^−1^ and expressed as nmol g^−1^ fresh weight (FW). This method provides a reliable estimate of lipid peroxidation and membrane damage induced by oxidative stress.

### 2.9 Hydrogen peroxide (H_2_O_2_)

Hydrogen peroxide content in plant leaves was quantified following the method of Velikova et al. (2000) using potassium iodide (KI)-based colorimetry. Approximately 0.5 g of fresh leaf tissue was homogenized in 5 mL of ice-cold 0.1% (w/v) trichloroacetic acid (TCA) using a pre-chilled mortar and pestle. The homogenate was centrifuged at 12,000 × g for 15 minutes at 4°C. A 0.5 mL aliquot of the resulting supernatant was mixed with 0.5 mL of 10 mM potassium phosphate buffer (pH 7.0) and 1 mL of 1 M potassium iodide (KI) solution. The mixture was incubated in the dark for 1 hour at room temperature. Absorbance was then measured at 390 nm using a UV–visible spectrophotometer (Shimadzu UV-1800, Japan). The H_2_O_2_ concentration was determined using a standard curve prepared with known concentrations of hydrogen peroxide and expressed as µmol g^−1^ fresh weight (FW). This method allows sensitive and accurate detection of H_2_O_2_ accumulation in response to oxidative stress.

### 2.10 Superoxide anion (O_2_•^−^)

The generation of superoxide anion (O_2_•^−^) in leaf tissues was assessed using the method of Elstner and Heupel (1976). Fresh leaf samples (0.5 g) were homogenized in 3 mL of 65 mM potassium phosphate buffer (pH 7.8) and centrifuged at 5,000 × g for 10 minutes at 4°C. The reaction mixture consisted of 1 mL of supernatant, 0.9 mL of 65 mM phosphate buffer (pH 7.8), and 0.1 mL of 10 mM hydroxylamine hydrochloride. After incubation at 25°C for 20 minutes, 1 mL of 17 mM sulfanilamide and 1 mL of 7 mM α-naphthylamine (both prepared in 2.5% acetic acid) were added to the mixture. The solution was further incubated for 20 minutes at 25 °C, and the absorbance was read at 530 nm using a UV–visible spectrophotometer. A standard curve of sodium nitrite was used to calculate the concentration of O_2_•^−^, which was expressed as nmol min^−1^ g^−1^ fresh weight (FW). This assay quantifies the nitrite produced by the reaction of hydroxylamine with superoxide radicals, serving as a reliable indicator of oxidative stress.

### 2.11 Electrolyte leakage

EL, an indicator of membrane damage under stress conditions, was measured following standard procedures using a conductivity meter (model DDS-307, INESA, China). Fresh leaf discs (0.2 g) were rinsed with deionized water to remove surface-adhered electrolytes and then immersed in 10 mL of deionized water in test tubes (Lutts et al., 1996). The samples were incubated at room temperature (25 °C) for 24 hours, after which the initial electrical conductivity of the bathing solution (C_1_) was recorded. Subsequently, the tubes were autoclaved at 121 °C for 20 minutes to completely lyse the cells and release all electrolytes. After cooling to room temperature, the final conductivity (C_2_) was measured. Electrolyte leakage was calculated as a percentage using the formula:.


$$\text{EL}\left(\%\right)=\left(C_{1}/C_{2}\right)\times 100$$

This method quantifies the degree of cellular membrane injury by assessing the extent of ion leakage into the surrounding medium.

### 2.12 Antioxidant enzyme assays

Fresh leaf tissues (0.5 g) were homogenized in 5 mL of 50 mM phosphate buffer (pH 7.0) containing 1% polyvinylpyrrolidone (PVP) and 0.1 mM ethylenediaminetetraacetic acid (EDTA) to stabilize enzymes and prevent phenolic oxidation. The inclusion of PVP ensured the removal of interfering phenolic compounds, while EDTA chelated divalent cations that could inactivate enzymes. The homogenate was centrifuged at 12,000 × g for 20 min at 4 °C, and the resulting supernatant was used immediately for the determination of enzymatic antioxidant activities. All assays were carried out under cold conditions to maintain enzyme stability and activity.

#### 2.12.1 Superoxide dismutase (SOD; EC 1.15.1.1)

The activity of SOD was assayed following the method of Beauchamp and Fridovich (1971), based on its ability to inhibit the photochemical reduction of nitroblue tetrazolium (NBT). In this assay, the reaction mixture containing riboflavin, methionine, NBT, and the enzyme extract was exposed to light, generating superoxide radicals that reduce NBT to blue formazan. SOD activity was quantified by measuring the decrease in absorbance at 560 nm due to inhibition of NBT reduction. One unit of SOD activity was defined as the amount of enzyme required to cause 50% inhibition of NBT photoreduction. SOD serves as the first line of defense against reactive oxygen species (ROS) by converting the superoxide anion (O_2_^−^•) into hydrogen peroxide (H_2_O_2_) and molecular oxygen, thus reducing oxidative stress in plant tissues.

#### 2.12.2 Catalase (CAT; EC 1.11.1.6)

The CAT activity was determined according to Aebi (1984) by monitoring the decomposition of H_2_O_2_ at 240 nm. The reaction mixture consisted of phosphate buffer and freshly prepared H_2_O_2_ solution, and the decline in absorbance corresponded to the breakdown of H_2_O_2_ into water and oxygen. One unit of CAT activity was expressed as the amount of enzyme decomposing 1 μmol of H_2_O_2_ per minute. CAT is a key antioxidant enzyme located in peroxisomes, responsible for detoxifying the H_2_O_2_ produced by SOD and other metabolic reactions, thereby preventing oxidative injury to cellular membranes and organelles.

#### 2.12.3 Peroxidase (POD; EC 1.11.1.7)

Peroxidase (POD) activity was estimated using the guaiacol oxidation method proposed by Chance and Maehly (1955). The reaction mixture contained phosphate buffer, guaiacol, and H_2_O_2_, and the formation of tetraguaiacol was monitored by an increase in absorbance at 470 nm. One unit of POD activity was defined as the amount of enzyme catalyzing the oxidation of 1 μmol of guaiacol per minute. Peroxidases are involved in detoxifying hydrogen peroxide and play crucial roles in lignin biosynthesis, suberization, and stress-induced defense mechanisms, contributing to cellular strengthening and ROS neutralization under salinity stress.

#### 2.12.4 Ascorbate peroxidase (APX; EC 1.11.1.11)

The activity of ascorbate peroxidase (APX) was assayed following Nakano and Asada (1981) by measuring the oxidation of ascorbate (AsA) at 290 nm. The reaction mixture included phosphate buffer, H_2_O_2_, and ascorbate as an electron donor. The decrease in absorbance corresponded to the consumption of AsA as it reduced H_2_O_2_ to water. One unit of APX activity represented the oxidation of 1 μmol of ascorbate per minute. APX is a vital enzyme of the ascorbate–glutathione (AsA–GSH) cycle, operating mainly in chloroplasts and cytosol, where it detoxifies H_2_O_2_ and preserves redox homeostasis during oxidative stress caused by salinity.

#### 2.12.5 Glutathione reductase (GR; EC 1.6.4.2)

Glutathione reductase (GR) activity was measured spectrophotometrically at 340 nm according to Foyer and Halliwell (1976), based on the oxidation of NADPH in the presence of oxidized glutathione (GSSG). The decrease in absorbance at 340 nm indicated NADPH consumption, which is stoichiometrically related to the regeneration of reduced glutathione (GSH). One unit of GR activity was expressed as the amount of enzyme oxidizing 1 μmol of NADPH per minute. GR plays a pivotal role in the maintenance of the cellular redox balance, as it regenerates GSH from GSSG, thereby sustaining the antioxidant capacity of the AsA–GSH cycle. All enzymatic activities were expressed as units (U) per milligram of protein, and protein concentration in the extracts was determined using the Bradford (1976) method with bovine serum albumin as a standard.

#### 2.12.6 Non-enzymatic antioxidants

The levels of ascorbate (AsA) and reduced glutathione (GSH) were quantified spectrophotometrically following Law et al. (1983) and Griffith (1980), respectively. For AsA determination, leaf extracts were reacted with dinitrophenylhydrazine after reduction with thiourea, and absorbance was read at 525 nm. GSH was determined using 5,5’-dithiobis-(2-nitrobenzoic acid) (DTNB), and the yellow-colored product was measured at 412 nm. The ratios of AsA/dehydroascorbate (DHA) and GSH/oxidized glutathione (GSSG) were calculated to evaluate the cellular redox state. A higher AsA/DHA and GSH/GSSG ratio reflects a more reducing cellular environment, indicating greater antioxidant capacity and resistance to oxidative damage under salinity stress.

#### 2.12.7 Statistical analysis

Data were analyzed using a two-way analysis of variance (ANOVA) for the factorial design [Salinity (3 levels) × GABA (3 levels)]. Means were compared using Fisher’s Least Significant Difference (LSD) test at a significance level of *p* ≤ 0.05. Prior to ANOVA, assumptions of normality and homogeneity of variance were assessed using the Shapiro–Wilk and Levene’s tests, respectively. Pearson correlation coefficients were calculated to examine relationships among growth, osmolyte, and antioxidant parameters.

## 3 Results

### 3.1 Growth responses to salinity and GABA treatments

The results presented in **Table 2** show that salinity stress markedly reduced plant growth attributes, including plant height, leaf number, leaf area, and both fresh and dry biomass of shoots and roots. Increasing NaCl concentration from 0 to 150 mM significantly (p ≤ 0.05) decreased all measured growth parameters, reflecting the detrimental effects of osmotic and ionic stress on plant vigor. In contrast, foliar application of γ-aminobutyric acid (GABA) significantly alleviated the inhibitory effects of salinity, with the most pronounced improvements observed under moderate (100 mM) and severe (150 mM) salt stress.

**Table 2:** Effect of salinity and γ-aminobutyric acid (GABA) on growth parameters of plants after six weeks of treatment.

Salinity (mM NaCl)	GABA (mM)	Plant Height (cm)	Leaf Number	Leaf Area (cm² plant^−1^)	Shoot FW (g)	Root FW (g)	Shoot DW (g)	Root DW (g)
0 (Control)	0 (G_0_)	36.8 ± 1.5 b	18.2 ± 0.8 b	158.6 ± 7.4 b	15.40 ± 0.65 b	5.20 ± 0.32 b	2.83 ± 0.12 b	0.92 ± 0.06 b
0	1 (G_1_)	39.5 ± 1.2 a	19.7 ± 0.9 a	172.3 ± 8.1 a	17.05 ± 0.58 a	5.76 ± 0.28 a	3.12 ± 0.15 a	1.05 ± 0.05 a
0	2 (G_2_)	38.9 ± 1.0 a	19.1 ± 1.1 a	170.1 ± 7.5 a	16.80 ± 0.66 a	5.70 ± 0.31 a	3.08 ± 0.13 a	1.02 ± 0.07 a
100	0	28.7 ± 1.3 e	14.6 ± 0.6 d	109.4 ± 5.6 d	10.15 ± 0.54 e	3.45 ± 0.25 d	1.92 ± 0.09 d	0.66 ± 0.05 d
100	1	32.5 ± 1.1 cd	16.7 ± 0.8 c	131.8 ± 6.2 c	12.90 ± 0.57 c	4.12 ± 0.27 c	2.38 ± 0.11 c	0.79 ± 0.06 c
100	2	33.8 ± 1.4 c	17.3 ± 0.9 bc	139.5 ± 6.9 bc	13.75 ± 0.60 c	4.38 ± 0.22 c	2.52 ± 0.10 c	0.84 ± 0.05 c
150	0	22.4 ± 1.2 f	11.2 ± 0.5 e	78.3 ± 4.3 e	7.05 ± 0.48 f	2.65 ± 0.19 e	1.36 ± 0.08 e	0.48 ± 0.04 e
150	1	25.6 ± 1.3 e	13.4 ± 0.7 d	95.6 ± 4.9 de	8.80 ± 0.53 de	3.15 ± 0.20 d	1.68 ± 0.09 d	0.60 ± 0.05 d
150	2	27.8 ± 1.1 de	14.1 ± 0.8 d	104.8 ± 5.1 d	9.60 ± 0.55 d	3.48 ± 0.23 d	1.86 ± 0.10 d	0.64 ± 0.05 d

Data are mean ± SE (n = 4). Different letters within each column indicate significant differences according to the *post hoc* test (p ≤ 0.05).

Under non-saline conditions, plants treated with 1 or 2 mM GABA (G_1_ and G_2_) exhibited slightly higher growth than untreated controls, indicating that GABA had a positive influence even in the absence of stress. The highest plant height (39.5 cm), leaf number (19.7), and leaf area (172.3 cm² plant^−1^) were recorded in plants treated with 1 mM GABA under control conditions (S_0_G_1_). However, exposure to 100 and 150 mM NaCl reduced these parameters by approximately 20–40% and 40–60%, respectively, relative to the control (S_0_G_0_).

Foliar application of GABA mitigated these reductions in a concentration-dependent manner. At 100 mM NaCl, plants treated with 1 and 2 mM GABA (S_1_G_1_ and S_1_G_2_) showed 13–18% higher plant height, ~20% greater leaf area, and ~25% higher shoot dry weight compared with the untreated salinity-stressed plants (S_1_G_0_). Similarly, under severe salinity (150 mM NaCl), GABA-treated plants exhibited substantial improvements in biomass accumulation. The shoot fresh weight increased from 7.05 g (S_2_G_0_) to 8.80–9.60 g (S_2_G_1_–S_2_G_2_), and root fresh weight rose from 2.65 g to 3.15–3.48 g, indicating enhanced water retention and root activity. Correspondingly, shoot and root dry weights were elevated by 20–30% in GABA-treated plants compared with untreated controls at the same salinity level.

### 3.2 Water status and membrane integrity

Salinity stress caused a significant reduction in RWC and MSI in plant leaves (**Table 3**). Under severe salinity conditions (150 mM NaCl), RWC and MSI decreased by 31% and 37%, respectively, compared to the control, indicating compromised water uptake and membrane integrity. The decline in RWC reflects osmotic stress-induced limitations on root water absorption and loss of cellular turgor, likely due to impaired hydraulic conductivity. Concurrently, the reduction in MSI suggests increased membrane damage, characterized by elevated electrolyte leakage resulting from lipid peroxidation and ionic toxicity.

**Table 3:** Effects of salinity and γ-aminobutyric acid (GABA) treatments on relative water content (RWC), membrane stability index (MSI), and osmolyte accumulation (proline, soluble sugars, and soluble protein) in plant leaves after six weeks of exposure.

Salinity (mM NaCl)	GABA (mM)	RWC (%)	MSI (%)	Proline (µmol g^−1^ FW)	Soluble sugars (mg g^−1^ FW)	Soluble protein (mg g^−1^ FW)
0 (Control)	0 (G_0_)	89.3 ± 1.4 a	85.6 ± 1.8 a	2.46 ± 0.15 d	10.8 ± 0.55 b	13.9 ± 0.63 a
0	1 (G_1_)	91.2 ± 1.1 a	87.4 ± 1.5 a	2.71 ± 0.18 cd	11.6 ± 0.64 a	14.5 ± 0.70 a
0	2 (G_2_)	90.7 ± 1.3 a	86.9 ± 1.6 a	2.63 ± 0.14 cd	11.3 ± 0.59 a	14.3 ± 0.66 a
100	0	74.8 ± 1.8 d	69.6 ± 1.9 d	5.35 ± 0.27 b	8.15 ± 0.47 d	9.56 ± 0.48 c
100	1	81.6 ± 1.6 b	78.2 ± 1.7 b	6.40 ± 0.33 a	9.50 ± 0.51 c	11.7 ± 0.53 b
100	2	83.2 ± 1.5 b	79.5 ± 1.8 b	6.25 ± 0.29 a	9.87 ± 0.55 bc	12.0 ± 0.55 b
150	0	61.4 ± 2.0 f	53.8 ± 2.1 f	7.42 ± 0.36 a	6.42 ± 0.43 e	7.22 ± 0.42 d
150	1	70.3 ± 1.8 e	63.6 ± 1.9 e	8.35 ± 0.40 a	7.55 ± 0.47 d	8.60 ± 0.44 c
150	2	72.8 ± 1.7 de	65.4 ± 1.8 de	8.10 ± 0.38 a	7.83 ± 0.46 d	8.90 ± 0.45 c

Data are mean ± SE (n = 4). Different letters within each column indicate significant differences according to the *post hoc* test (p ≤ 0.05).

However, foliar application of GABA effectively alleviated these adverse effects. Under both moderate (100 mM) and severe (150 mM) salinity, plants treated with 1–2 mM GABA exhibited significantly higher RWC and MSI values than untreated stressed plants. The improvement was particularly notable in the S_2_G_1_ treatment group, where RWC increased from 61.4% to 70.3%, and MSI rose from 53.8% to 63.6%, indicating enhanced cellular hydration and membrane protection.

### 3.3 Osmolyte accumulation and metabolic adjustment

Accumulation of compatible solutes, including proline, soluble sugars, and soluble proteins, is a common adaptive response to salinity. In the present study, proline content increased more than threefold under 150 mM NaCl compared with the control (from 2.46 to 7.42 µmol g^−1^ FW), indicating its role in osmotic regulation and ROS detoxification. Notably, GABA-treated plants accumulated even higher levels of proline, reaching up to 8.35 µmol g^−1^ FW in S_2_G_1_ plants. This result suggests that GABA enhances proline biosynthesis, likely by stimulating pyrroline-5-carboxylate synthetase (P5CS) and inhibiting proline dehydrogenase, key enzymes regulating proline metabolism. Similarly, soluble sugars and proteins increased with salinity, reflecting their roles in osmotic balance, stabilization of macromolecules, and maintenance of cell turgor.

### 3.4 Oxidative stress markers and membrane damage

Exposure to salinity led to a pronounced increase in MDA, H_2_O_2_, O_2_•^−^, and EL (**Table 4**). These indicators reflect lipid peroxidation and oxidative damage caused by the overproduction of reactive ROS under ionic and osmotic stress. The MDA level nearly tripled in severely stressed plants (from 3.42 to 9.83 nmol g^−1^ FW), confirming enhanced membrane peroxidation. Similarly, H_2_O_2_ and O_2_•^−^ levels increased by more than twofold, while EL rose to 46.2%, indicating severe disruption of plasma membrane integrity. GABA treatment substantially reduced these oxidative markers, highlighting its antioxidant role. Under 150 mM NaCl, MDA, H_2_O_2_, and O_2_•^−^ levels were lowered by approximately 30–40% in GABA-treated plants compared with untreated ones. This reduction is consistent with the activation of enzymatic antioxidants such as SOD, CAT, APX, and GR, and the maintenance of non-enzymatic antioxidants like ascorbate and glutathione.

**Table 4:** Effects of salinity and γ-aminobutyric acid (GABA) treatments on oxidative stress indicators, including malondialdehyde (MDA), hydrogen peroxide (H_2_O_2_), superoxide (O_2_^−^), and electrolyte leakage in plant leaves after six weeks of exposure.

Salinity (mM NaCl)	GABA (mM)	Malondialdehyde (nmol g^−1^ FW)	Hydrogen peroxide (µmol g^−1^ FW)	Superoxide (nmol g^−1^ FW)	Electric leakage (%)
0 (Control)	0 (G_0_)	3.42 ± 0.19 e	2.18 ± 0.14 e	2.75 ± 0.17 d	14.6 ± 0.8 e
0	1 (G_1_)	3.16 ± 0.16 e	2.05 ± 0.12 e	2.52 ± 0.14 d	13.4 ± 0.7 e
0	2 (G_2_)	3.22 ± 0.18 e	2.09 ± 0.13 e	2.58 ± 0.16 d	13.8 ± 0.9 e
100	0	7.95 ± 0.32 b	5.12 ± 0.27 b	6.75 ± 0.35 b	32.8 ± 1.4 b
100	1	5.60 ± 0.27 c	3.84 ± 0.20 c	4.82 ± 0.29 c	25.5 ± 1.2 c
100	2	5.42 ± 0.25 c	3.70 ± 0.18 c	4.66 ± 0.26 c	23.9 ± 1.1 c
150	0	9.83 ± 0.39 a	6.80 ± 0.32 a	8.16 ± 0.44 a	46.2 ± 1.8 a
150	1	7.06 ± 0.31 b	4.68 ± 0.24 b	6.05 ± 0.33 b	34.7 ± 1.5 b
150	2	6.85 ± 0.29 b	4.52 ± 0.22 b	5.88 ± 0.30 b	32.4 ± 1.4 b

Data are mean ± SE (n = 4). Different letters within each column indicate significant differences according to the *post hoc* test (p ≤ 0.05).

### 3.5 Antioxidant enzyme activities

The activities of SOD, CAT, POD, APX, and GR were markedly influenced by salinity and GABA treatments (**Table 5**). Salinity stress alone significantly enhanced the activities of all antioxidant enzymes compared with the non-saline control, reflecting a stress-induced activation of the antioxidant defense system in response to elevated reactive oxygen species (ROS) production. However, plants exposed to high salinity (150 mM NaCl) without GABA exhibited a partial decline in enzyme activities relative to those under moderate salinity (100 mM), suggesting oxidative damage and reduced enzymatic efficiency at severe stress levels.

**Table 5:** Effect of salinity and γ-aminobutyric acid (GABA) on antioxidant enzyme activities in leaves after six weeks of treatment.

Salinity (mM NaCl)	GABA (mM)	Superoxide dismutase (SOD)	Catalase (CAT)	Peroxidase (POD)	Ascorbate peroxidase (APX)	Glutathione reductase (GR)
		(U mg^−1^ protein)				
0 (Control)	0 (G_0_)	36.8 ± 1.5 d	22.4 ± 1.2 d	18.6 ± 0.9 d	8.9 ± 0.6 c	6.8 ± 0.4 c
0	1 (G_1_)	41.5 ± 1.4 c	25.8 ± 1.3 c	21.4 ± 1.1 c	10.5 ± 0.7 bc	7.6 ± 0.5 bc
0	2 (G_2_)	42.7 ± 1.3 c	26.5 ± 1.1 c	22.0 ± 1.0 c	10.9 ± 0.6 bc	7.8 ± 0.4 bc
100	0	49.6 ± 1.8 b	31.7 ± 1.5 b	27.8 ± 1.2 b	12.4 ± 0.7 b	8.9 ± 0.5 b
100	1	56.8 ± 1.6 a	36.9 ± 1.6 a	32.5 ± 1.3 a	15.6 ± 0.8 a	10.2 ± 0.6 a
100	2	55.3 ± 1.5 a	35.8 ± 1.4 a	31.8 ± 1.1 a	15.1 ± 0.7 a	9.8 ± 0.5 a
150	0	44.7 ± 1.7 c	28.4 ± 1.4 c	24.6 ± 1.1 c	11.0 ± 0.6 bc	7.2 ± 0.4 bc
150	1	53.6 ± 1.6 a	34.5 ± 1.3 a	29.9 ± 1.2 a	14.4 ± 0.7 a	9.1 ± 0.5 a
150	2	52.3 ± 1.5 a	33.8 ± 1.2 a	29.1 ± 1.1 a	14.0 ± 0.6 a	8.8 ± 0.4 a

Data are mean ± SE (n = 4). Different letters within each column indicate significant differences according to the *post hoc* test (p ≤ 0.05).

Under non-saline conditions, GABA-treated plants (1 and 2 mM) displayed slightly higher enzyme activities than untreated controls, indicating that GABA itself may prime the antioxidant machinery even in the absence of stress. The most pronounced enhancement, however, occurred under saline conditions. At 100 mM NaCl, foliar application of 1 mM GABA significantly increased SOD, CAT, and POD activities by approximately 15–25% relative to untreated stressed plants. Similarly, APX and GR activities were elevated by 25–30%, indicating the stimulation of the ascorbate–glutathione (AsA–GSH) cycle. The improvement was slightly less pronounced with 2 mM GABA, suggesting that 1 mM was the optimal concentration for enzyme activation.

At severe salinity (150 mM NaCl), exogenous GABA maintained high antioxidant enzyme activities, preventing the decline observed in untreated plants. For instance, SOD activity increased from 44.7 to 53.6 U mg^−1^ protein, while CAT rose from 28.4 to 34.5 U mg^−1^ protein under GABA treatment. Concurrent increases in POD, APX, and GR further confirmed GABA’s capacity to reinforce the enzymatic antioxidant network under stress. This enhancement in enzymatic defense was accompanied by a significant reduction in oxidative stress indicators (MDA, H_2_O_2_, and O_2_•^−^ levels; **Table 4**), implying that GABA-mediated upregulation of antioxidant enzymes effectively limited ROS accumulation and lipid peroxidation.

The increase in SOD activity under GABA application highlights its primary role in converting O_2_•^−^ into H_2_O_2_, which is then detoxified by CAT, POD, and APX. The elevated activities of APX and GR further demonstrate enhanced operation of the AsA–GSH cycle, ensuring continuous regeneration of ascorbate and glutathione to sustain redox homeostasis.

### 3.6 Non-enzymatic antioxidants

**Figure 1:**
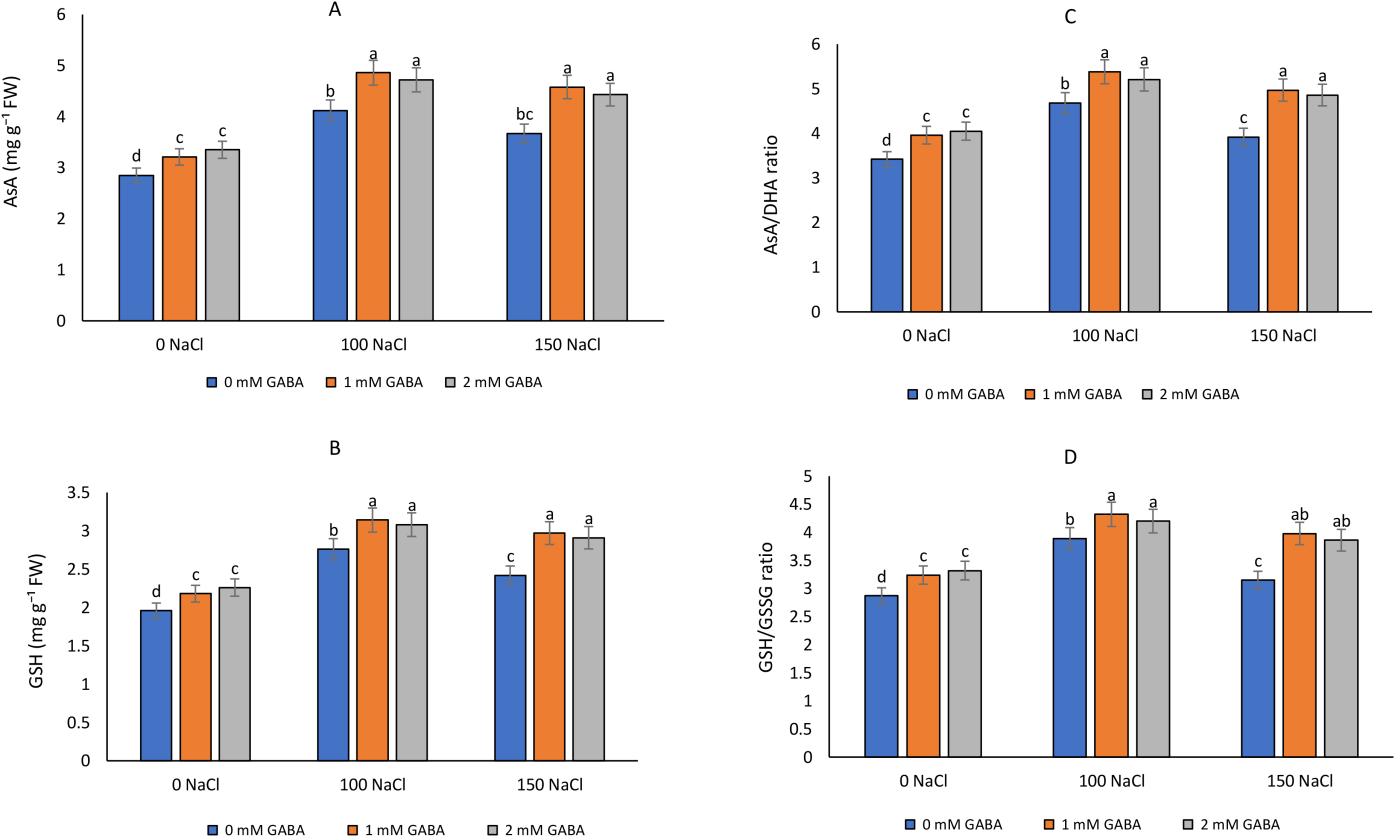
Effects of salinity (0 mM NaCl = control; 100 mM NaCl = moderate salinity stress; 150 mM NaCl = severe salinity stress) and γ-aminobutyric acid (GABA; 0, 1, and 2 mM) treatments on non-enzymatic antioxidant systems in plant leaves after six weeks of exposure. Parameters measured include: **(A)** ascorbate (AsA), **(B)** reduced glutathione (GSH), **(C)** AsA to dehydroascorbate (AsA/DHA) ratio, and **(D)** GSH to oxidized glutathione (GSH/GSSG) ratio. Values represent means ± standard error (SE) (n = 4). Different lowercase letters above bars indicate statistically significant differences among treatment means based on *post hoc* tests (*p* ≤ 0.05).

Salinity stress significantly altered the levels of non-enzymatic antioxidants, including AsA, GSH, and their redox ratios (AsA/DHA and GSH/GSSG) (**Figures 1A–D**). The marked reduction in these parameters under increasing NaCl concentration reflected a disturbance in redox homeostasis and the depletion of cellular reducing power under oxidative stress. In contrast, exogenous GABA application enhanced both the absolute antioxidant pool and the redox ratios, suggesting its role in maintaining a more reduced cellular environment conducive to stress tolerance.

Under non-saline conditions, AsA and GSH contents were high, and their corresponding redox ratios remained stable, indicating a well-balanced antioxidant status. Exposure to 100 and 150 mM NaCl caused a progressive decline in AsA and GSH levels by approximately 15–30% compared with the control. This reduction corresponded with decreased AsA/DHA and GSH/GSSG ratios, indicating oxidation of the antioxidant pools due to excess reactive oxygen species (ROS) accumulation. However, foliar application of 1 mM GABA significantly counteracted this effect. At 100 mM NaCl, GABA-treated plants showed 20–25% higher AsA and 30% higher GSH levels than untreated plants, along with elevated AsA/DHA and GSH/GSSG ratios, demonstrating enhanced redox buffering capacity. The 2 mM GABA treatment also improved antioxidant status, though the effects were slightly lower than the 1 mM treatment, suggesting an optimal concentration threshold for redox regulation.

At severe salinity (150 mM NaCl), GABA application maintained AsA and GSH contents close to those of plants under moderate stress, indicating effective mitigation of oxidative damage. The AsA/DHA ratio increased from 3.2 in untreated to 3.9 in GABA-treated plants, while the GSH/GSSG ratio improved from 3.5 to 4.3, implying restoration of cellular redox equilibrium.

### 3.7 Macronutrients uptake

Salinity stress significantly decreased the uptake of macronutrients (N, P, K, Ca) while markedly increasing Na^+^ accumulation, leading to a sharp rise in the Na^+^/K^+^ ratio (**Table 6**). Plants subjected to 150 mM NaCl exhibited the lowest levels of N (20.6 mg g^−1^ DW), P (3.02 mg g^−1^ DW), and K (25.3 mg g^−1^ DW), accompanied by the highest Na^+^ concentration (38.2 mg g^−1^ DW) and Na^+^/K^+^ ratio (1.51). This ionic imbalance reflects severe disruption of nutrient transport and membrane selectivity under salt stress. Exogenous GABA markedly improved nutrient uptake under both moderate and severe salinity. At 100 mM NaCl, foliar GABA application (1 mM) increased N, P, K, and Ca contents by 10–15% compared with untreated plants, while reducing Na^+^ accumulation by nearly 20%. A similar pattern was observed under 150 mM NaCl, where GABA-treated plants maintained higher macronutrient levels and a substantially lower Na^+^/K^+^ ratio (≈ 1.1 *vs*. 1.5 in untreated plants), indicating enhanced ion homeostasis and reduced Na^+^ toxicity.

**Table 6:** Effect of salinity and γ-aminobutyric acid (GABA) on mineral nutrient uptake (N, P, K, Ca, Na) and Na^+^/K^+^ ratio in plants after six weeks of treatment.

Salinity (mM NaCl)	GABA (mM)	Nitrogen (N)	Phosphorus (P)	Potassium (K)	Calcium (Ca)	Sodium (Na)	Na^+^/K^+^
		(mg g^−1^ DW)					
0 (Control)	0 (G_0_)	31.6 ± 1.2 b	5.18 ± 0.24 b	42.8 ± 1.4 b	14.2 ± 0.6 a	10.6 ± 0.5 e	0.25 ± 0.02 e
0	1 (G_1_)	33.9 ± 1.0 a	5.65 ± 0.22 a	44.7 ± 1.5 a	14.6 ± 0.5 a	10.0 ± 0.4 e	0.22 ± 0.02 e
0	2 (G_2_)	33.2 ± 1.1 a	5.49 ± 0.25 a	43.9 ± 1.3 a	14.4 ± 0.6 a	10.3 ± 0.4 e	0.23 ± 0.02 e
100	0	25.4 ± 1.0 e	3.92 ± 0.19 d	31.8 ± 1.2 d	11.2 ± 0.4 c	26.5 ± 1.2 c	0.83 ± 0.05 c
100	1	28.6 ± 1.1 c	4.46 ± 0.21 c	35.5 ± 1.3 c	12.3 ± 0.5 b	22.2 ± 1.1 d	0.63 ± 0.04 d
100	2	27.8 ± 1.2 cd	4.31 ± 0.20 cd	34.6 ± 1.2 c	12.0 ± 0.5 b	23.4 ± 1.0 cd	0.68 ± 0.04 cd
150	0	20.6 ± 0.9 f	3.02 ± 0.18 e	25.3 ± 1.1 e	9.6 ± 0.4 d	38.2 ± 1.6 a	1.51 ± 0.08 a
150	1	23.8 ± 1.0 e	3.58 ± 0.17 d	28.4 ± 1.2 d	10.8 ± 0.5 c	31.4 ± 1.3 b	1.11 ± 0.06 b
150	2	22.5 ± 1.0 ef	3.36 ± 0.18 d	27.2 ± 1.1 d	10.4 ± 0.5 c	32.8 ± 1.4 b	1.21 ± 0.07 b

Data are mean ± SE (n = 4). Different letters within each column indicate significant differences according to the *post hoc* test (p ≤ 0.05).

### 3.8 Micronutrients uptake

Salinity stress caused a significant reduction in the uptake of all tested micronutrients (Fe, Mn, Zn, and Cu) in plant tissues, and this decline became more pronounced as NaCl concentration increased (**Table 7**). Under severe salinity (150 mM NaCl), Fe, Mn, Zn, and Cu contents dropped by 39%, 38%, 43%, and 38%, respectively, compared with control plants. This pattern reflects the detrimental effects of Na^+^ accumulation on the rhizosphere nutrient availability and root ion transport systems.

**Table 7:** Effect of salinity and γ-aminobutyric acid (GABA) on micronutrient uptake (Fe, Mn, Zn, Cu) in plants after six weeks of treatment.

Salinity (mM NaCl)	GABA (mM)	Iron (Fe)	Manganese (Mn)	Zinc (Zn)	Copper (Cu)
		(µg g^−1^ DW)			
0 (Control)	0 (G_0_)	185.6 ± 6.8 b	112.4 ± 5.3 b	68.2 ± 2.8 b	21.6 ± 1.0 b
0	1 (G_1_)	198.7 ± 7.1 a	120.5 ± 5.5 a	72.9 ± 2.9 a	23.2 ± 1.1 a
0	2 (G_2_)	193.2 ± 6.9 a	118.6 ± 5.2 a	71.4 ± 2.8 a	22.9 ± 1.0 a
100	0	143.8 ± 5.9 d	86.4 ± 4.1 d	49.7 ± 2.2 d	17.3 ± 0.8 c
100	1	164.5 ± 6.2 c	98.2 ± 4.5 c	57.9 ± 2.4 c	19.6 ± 0.9 b
100	2	158.2 ± 6.0 c	94.3 ± 4.3 c	55.6 ± 2.3 c	18.8 ± 0.9 bc
150	0	112.7 ± 5.1 f	69.8 ± 3.8 e	38.6 ± 1.9 e	13.5 ± 0.7 d
150	1	131.5 ± 5.6 e	80.7 ± 3.9 d	45.8 ± 2.0 d	15.9 ± 0.8 cd
150	2	126.3 ± 5.4 e	78.4 ± 3.8 d	43.9 ± 2.0 d	15.2 ± 0.8 cd

Data are mean ± SE (n = 4). Different letters within each column indicate significant differences according to the *post hoc* test (p ≤ 0.05).

Application of GABA alleviated the salinity-induced micronutrient deficiency. At 100 mM NaCl, foliar spraying with 1 mM GABA increased Fe, Mn, Zn, and Cu uptake by 14–18% relative to the untreated plants. Similarly, under 150 mM NaCl, GABA-treated plants accumulated higher levels of Fe (131.5 µg g^−1^ DW) and Mn (80.7 µg g^−1^ DW) compared with untreated plants (112.7 and 69.8 µg g^−1^ DW, respectively). The moderate GABA dose (1 mM) consistently showed superior performance compared to 2 mM, indicating an optimal physiological concentration for nutrient uptake enhancement.

### 3.9 Integrated heatmap analysis of physiological, biochemical, and nutritional responses

**Figure 2:**
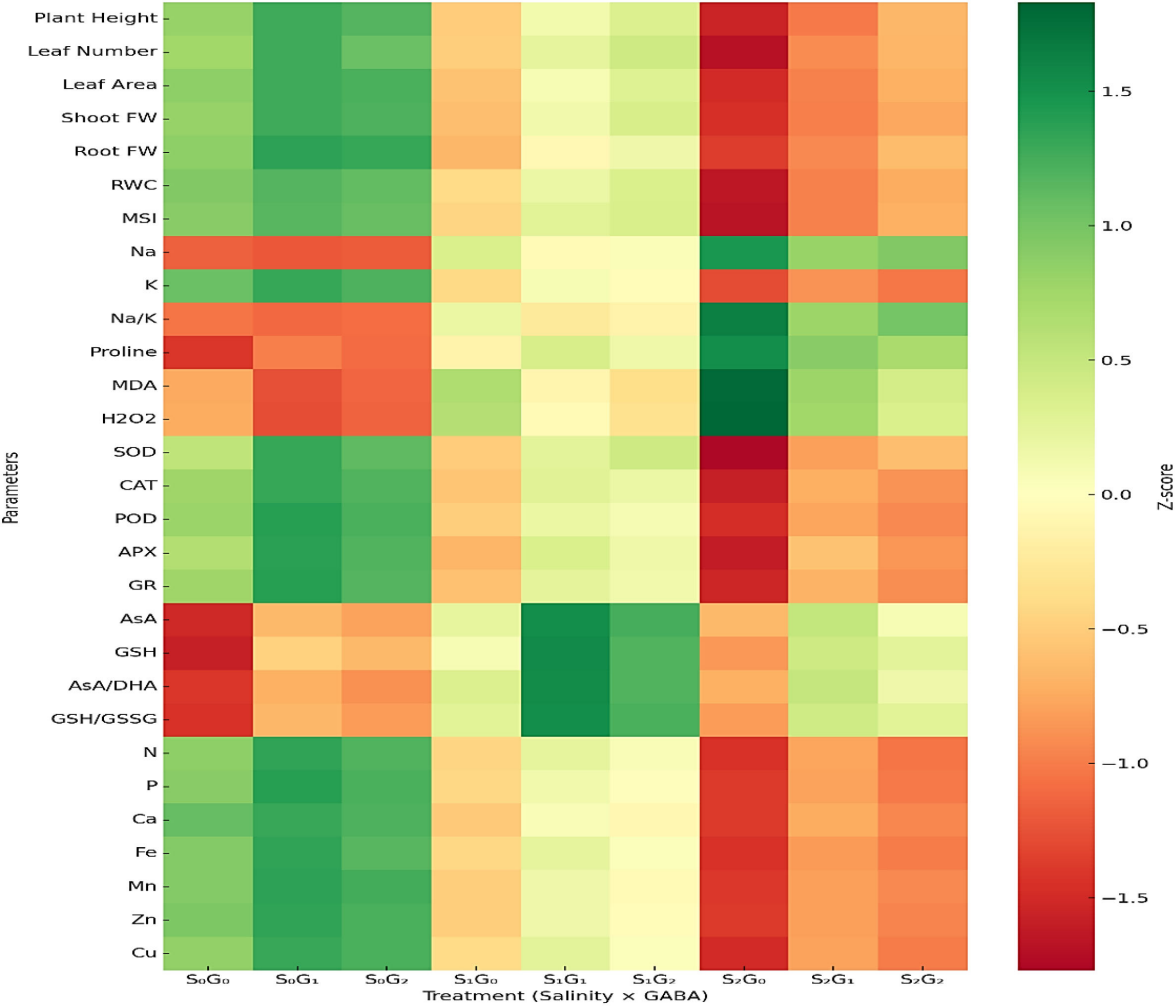
Integrated heatmap illustrating the effects of salinity and γ-aminobutyric acid (GABA) on plant growth, physiological, biochemical, and nutrient uptake parameters after six weeks of treatment. Each column represents a combined salinity × GABA treatment (S_0_G_0_, S_0_G_1_, S_0_G_2_, S_1_G_0_, S_1_G_1_, S_1_G_2_, S_2_G_0_, S_2_G_1_, S_2_G_2_), where S_0_ = 0 mM NaCl (control), S_1_ = 100 mM NaCl (moderate stress), and S_2_ = 150 mM NaCl (severe stress); G_0_ = 0 mM, G_1_ = 1 mM, and G_2_ = 2 mM GABA. Colors represent Z-score–normalized values (green = higher, red = lower relative to the mean). Plant Height, LN, Leaf Number; LA, Leaf Area; SFW, Shoot Fresh Weight; RFW, Root Fresh Weight; RWC, Relative Water Content; MSI, Membrane Stability Index; Na^+^, Sodium; K^+^, Potassium; Na^+^/K^+^, Sodium/Potassium ratio; Pro, Proline; MDA, Malondialdehyde; H_2_O_2_, Hydrogen Peroxide; SOD, Superoxide Dismutase; CAT, Catalase; POD, Peroxidase; APX, Ascorbate Peroxidase; GR, Glutathione Reductase; AsA, Ascorbate; GSH, Reduced Glutathione; AsA/DHA, Ascorbate/Dehydroascorbate ratio; GSH/GSSG, Glutathione/Oxidized Glutathione ratio; N, Nitrogen; P, Phosphorus; Ca, Calcium; Fe, Iron; Mn, Manganese; Zn, Zinc; and Cu, Copper.

The integrated heatmap (**Figure 2**) provides a comprehensive overview of how salinity and GABA treatments modulated growth, physiological, biochemical, and nutrient uptake traits in plants after six weeks of exposure. Clear clustering patterns were observed among treatments, reflecting a coordinated response of metabolic and physiological systems to both salinity stress and GABA application.

Under salinity stress alone, plants exhibited pronounced declines in growth attributes (plant height, leaf number, leaf area, and biomass) and water status indicators (RWC, MSI), accompanied by elevated oxidative stress markers such as MDA and H_2_O_2_. The Na^+^ and Na^+^/K^+^ ratio sharply increased under these treatments, indicating ion toxicity and disrupted membrane transport. Concurrently, activities of antioxidant enzymes and levels of non-enzymatic antioxidants (AsA, GSH) decreased relative to the control, signifying oxidative imbalance and reduced detoxification capacity.

By contrast, plants treated with exogenous GABA, particularly at 1 mM (G_1_), exhibited a clear shift toward green zones in the heatmap, denoting overall improvement across multiple parameters. GABA treatment restored water balance and membrane integrity (higher RWC and MSI) while reducing MDA, H_2_O_2_, and Na^+^ accumulation. Simultaneously, it enhanced both enzymatic and non-enzymatic antioxidant defenses, reflected by higher SOD, CAT, POD, APX, GR, AsA, and GSH levels and more favorable AsA/DHA and GSH/GSSG ratios. These enhancements are indicative of strengthened redox homeostasis and improved ROS-scavenging capacity under saline conditions.

Nutrient uptake patterns followed a similar trend. The uptake of macronutrients (N, P, K, Ca) and micronutrients (Fe, Mn, Zn, Cu) decreased sharply with increasing salinity, while GABA application significantly mitigated these declines. This restoration suggests GABA-mediated stabilization of membrane transporters and improved root metabolic activity under ionic stress. The clustering of S_0_G_1_ and S_1_G_1_ treatments near each other in the normalized matrix implies that moderate salinity combined with optimal GABA application elicited physiological performance comparable to that of unstressed plants.

## 4 Discussion

Salinity stress significantly hampered plant growth in our study, as evidenced by reduced height, leaf number, and biomass production. This growth inhibition is a well-documented effect of salt-induced osmotic and ionic stress on plants (Qian et al., 2024). Importantly, exogenous GABA application markedly alleviated these growth reductions. GABA-treated plants under moderate (100 mM NaCl) and severe (150 mM NaCl) stress maintained greater shoot and root biomass compared to untreated controls, reflecting improved stress tolerance. These findings align with reports in other crops; for example, Badr et al. (2024) observed that GABA spraying promoted root and shoot growth in salt-stressed wheat seedlings. Likewise, Qian et al. (2024) found that exogenous GABA improved overall growth and biomass in salt-exposed soybean plants (Badr et al., 2024). The growth-promoting effect of GABA under saline conditions may stem from its role in mitigating ion toxicity and oxidative damage (discussed below), thereby allowing plants to sustain normal growth processes. In addition, GABA itself might act as a signaling molecule to modulate growth-related hormones and gene expression, priming plants to better withstand stress (Badr et al., 2024). The ability of GABA to preserve plant vigor under salt stress highlights its potential as an effective agronomic supplement for improving crop resilience in saline soils.

A key aspect of salinity stress is osmotic dehydration, which leads to reduced tissue water content and loss of turgor. In our study, RWC declined sharply under high salinity (up to ~30% reduction at 150 mM NaCl), accompanied by a drop in MSI and increased electrolyte leakage. These changes indicate that salt-stressed plants experienced difficulty in water uptake and suffered membrane lipid peroxidation and damage. Foliar GABA application significantly ameliorated these effects: GABA-treated plants maintained higher RWC and MSI than untreated plants at equivalent salt levels. Notably, 1 mM GABA under severe stress raised RWC by ~9 percentage points and improved MSI by ~10 percentage points relative to non-GABA controls, suggesting improved cellular hydration and membrane protection. This protective role of GABA is consistent with observations in other systems. For instance, GABA-treated wheat plants under salt stress showed enhanced RWC and minimized membrane injury, indicating better water status and stability of cell membranes (Yousaf et al., 2025). By maintaining higher leaf hydration, GABA likely helps preserve stomatal function and photosynthesis under stress, as also reported by Badr et al. (2024). The mechanism may involve GABA-induced accumulation of osmolytes (see below) that lower cellular osmotic potential, thereby improving water retention. GABA might also regulate aquaporin channels or root hydraulic conductance, facilitating water uptake under saline conditions (Su et al., 2019). Furthermore, the improved membrane integrity (higher MSI, lower leakage) in GABA-treated plants can be attributed to GABA’s capacity to strengthen antioxidant defenses, which prevents excessive lipid peroxidation of membranes (Chen et al., 2024). Altogether, our results demonstrate that exogenous GABA helps plants maintain better water balance and membrane structure integrity during salt stress, a crucial aspect of salinity tolerance.

The accumulation of compatible osmolytes is a common adaptive strategy for plants under salt stress, aiding in osmotic adjustment and protection of cellular components. In our study, leaf proline content increased more than threefold under 150 mM NaCl compared to non-stressed conditions, reflecting an intrinsic stress response. Notably, GABA treatments further boosted osmolyte levels beyond the salt-induced accumulation. GABA-sprayed plants (especially at 1 mM) accumulated significantly higher proline (up to ~12% more than untreated at 150 mM NaCl), as well as increased soluble sugars and soluble proteins in leaves. These osmolytes help to maintain cell turgor, stabilize proteins and membranes, and scavenge ROS under stress (Qian et al., 2024). Our findings concur with previous reports that exogenous GABA enhances osmolyte accumulation under abiotic stress. For example, Qian et al. (2024) reported that GABA-treated soybean seedlings under salinity accumulated higher levels of proline, glycine betaine, soluble sugars, and soluble proteins than untreated plants. The GABA-mediated increase in proline may result from upregulation of proline biosynthetic enzymes (e.g. pyrroline-5-carboxylate synthetase) and/or inhibition of proline degradation, as has been suggested in other studies (Qian et al., 2024; Zarbakhsh et al., 2025). Proline, in particular, not only contributes to osmotic adjustment but also stabilizes sub-cellular structures and buffers redox status by interacting with ROS (Hayat et al., 2012). The higher soluble sugar content in GABA-treated plants implies that GABA might influence carbon metabolism or source-sink relations to channel more sugars into osmoprotection (Li et al., 2021). Similarly, elevated soluble protein levels could indicate stress-responsive proteins (such as late embryogenesis abundant proteins or dehydrins) are more abundantly synthesized when GABA is applied. These compatible solutes collectively improve stress tolerance by maintaining enzyme function and hydration at the cellular level (Feng et al., 2024). Therefore, our results support the notion that GABA induces a metabolic adjustment towards greater osmolyte accumulation, which is instrumental in mitigating the osmotic stress imposed by salinity.

Salt stress often leads to overproduction of ROS like O_2_·^−^ and H_2_O_2_, which can cause oxidative damage to lipids, proteins, and nucleic acids (Qian et al., 2024). In our experiment, salinity-stressed plants showed sharply elevated levels of MDA, a lipid peroxidation marker, H_2_O_2_, and superoxide anion, along with increased electrolyte leakage all indicators of oxidative stress and membrane damage. Exogenous GABA markedly reduced these oxidative stress markers. Under 150 mM NaCl, GABA-treated plants had ~30–40% lower MDA and H_2_O_2_ concentrations and significantly less electrolyte leakage compared to untreated plants at the same salinity. This clearly demonstrates that GABA alleviated ROS-induced injury in the salt-stressed plants. Such protective effects are in agreement with numerous studies highlighting GABA’s antioxidative role. For instance, Chen et al. (2024) showed that rice seedlings with higher endogenous GABA levels accumulated less H_2_O_2_ and MDA under salt stress, whereas GABA-deficient mutants suffered greater oxidative damage (Chen et al., 2024). Similarly, exogenous GABA application in soybean was found to lower H_2_O_2_ and MDA content under saline conditions, leading to improved membrane stability (Qian et al., 2024). The reduction in ROS and MDA suggests that GABA either limits ROS generation or enhances their scavenging (or both). In GABA-treated plants, we observed a substantial upregulation of antioxidant defense systems (enzymatic and non-enzymatic), which likely explains the lower oxidative burden.

Salinity stress alone triggered an increase in activities of major antioxidant enzymes in our plants, particularly at 100 mM NaCl, as the plants attempted to detoxify excess ROS. However, at 150 mM NaCl, some enzyme activities in untreated plants declined, possibly due to enzyme inactivation or oxidative damage at extreme stress levels. GABA application not only prevented this decline but significantly boosted enzyme activities above the native stress response. Notably, 1 mM GABA under high salinity increased SOD, CAT, and POD activities by ~15–20% and APX and GR by ~20–30% compared to untreated controls. This suggests that GABA primed the antioxidant enzymatic machinery to be more robust in the face of stress. Enhanced activity of SOD is crucial as it dismutates superoxide radicals to H_2_O_2_, which is then neutralized by CAT and APX; the latter, along with GR, is part of the AsA–GSH cycle that regenerates antioxidants (Qian et al., 2024). Our results are in line with earlier findings that GABA can activate antioxidant enzymes under stress. For example, in salt-stressed wheat, GABA treatments were reported to activate enzymes like SOD and CAT, contributing to improved ROS scavenging (Badr et al., 2024; Dabravolski and Isayenkov, 2023). Qian et al. (2024) similarly noted an overall enhancement of enzymatic antioxidant activity in GABA-treated soybeans under salinity. By sustaining higher antioxidant enzyme levels, GABA-treated plants can more effectively keep ROS levels in check, preventing oxidative damage to membranes (hence lower MDA and electrolyte leakage observed) (Chen et al., 2024). This fortified enzymatic defense is a key component of the GABA-induced tolerance mechanism.

In addition to enzymes, plants rely on small-molecule antioxidants such as AsA and GSH to neutralize ROS and maintain redox homeostasis. Salinity in our study caused a notable depletion of AsA and GSH pools (accompanied by increased oxidized forms DHA and GSSG), indicating that the oxidative stress was consuming these antioxidants and shifting the cellular redox balance toward a more oxidized state. Foliar GABA applications helped preserve these non-enzymatic antioxidant pools. GABA-treated plants under salt stress showed higher AsA and GSH contents and more favorable AsA/DHA and GSH/GSSG ratios than untreated stressed plants, meaning a greater proportion of these antioxidants remained in their reduced, active forms. This suggests that GABA either directly or indirectly aided the regeneration of AsA and GSH, keeping the AsA–GSH cycle operational. Our findings mirror those of previous studies: exogenous GABA has been reported to elevate AsA and GSH levels in stressed plants, thereby bolstering their redox buffering capacity (Ullah et al., 2023). For instance, Qian et al. (2024) observed that GABA supplementation increased the content of AsA and GSH in salt-stressed soybean, concomitant with lower levels of oxidized DHA and GSSG (higher AsA/DHA, GSH/GSSG ratios. Maintaining a higher reduced AsA and GSH pool ensures continuous detoxification of ROS via the AsA–GSH cycle, working in concert with the enzymatic antioxidants APX and GR (Gao et al., 2024). Thus, GABA’s enhancement of both enzymatic and non-enzymatic antioxidant defenses creates a synergistic protective effect, effectively mitigating oxidative stress damage under salinity.

Excessive accumulation of Na^+^ and the resultant imbalance with K^+^ is a primary cause of ion toxicity under salt stress (Weisany et al., 2011). In our untreated plants, 150 mM NaCl led to a dramatic rise in tissue Na^+^ content and a Na^+^/K^+^ ratio approximately 1.5-fold higher than control, alongside marked declines in essential macronutrients like K, Ca, N, and P. This indicates that high external NaCl not only introduces toxic Na^+^ into plant tissues but also impairs the uptake of other nutrients (due to competitive uptake inhibition and general membrane transporter dysfunction) (Alharbi et al., 2022). Foliar GABA application significantly improved ion homeostasis in salt-stressed plants. GABA-treated plants accumulated less Na^+^ and maintained a much lower Na^+^/K^+^ ratio than untreated plants at the same salinity level, while showing higher concentrations of K^+^ and other nutrients in their shoots (Qian et al., 2024). For instance, at 150 mM NaCl, GABA treatments reduced Na^+^ content in shoots by nearly 20% and improved K^+^ levels by ~10–15%, thereby restoring a more balanced ionic ratio. Additionally, GABA-fed plants had enhanced uptake of Ca^2+^ and Mg^2+^ (important for membrane stabilization and enzyme function), as well as better nitrogen and phosphorus status than non-GABA plants under salt stress. This broad improvement in nutrient profile suggests that GABA positively influences root ion transport processes and nutrient acquisition capacity under saline conditions.

Our results are corroborated by recent studies demonstrating GABA’s role in modulating ion transport and distribution under salt stress. Su et al. (2019) provided compelling evidence in Arabidopsis that GABA operates upstream of the plasma membrane H^+^-ATPase pump to maintain membrane potential stability, which in turn helps prevent K^+^ loss from roots and reduces Na^+^ influx. The GABA-over accumulating Arabidopsis mutants in their study showed lower net Na^+^ uptake and enhanced K^+^ retention, partly due to higher expression of the SOS1 (plasma membrane Na^+^ exporter) Na^+^/H^+^ antiporter and vacuolar Na^+^ sequestration protein (NHX1) tonoplast Na^+^/H^+^ exchanger that extrude Na^+^ out of cytosol or sequester it into vacuoles (Su et al., 2019). Consistently, we observed that GABA-treated plants maintained a much healthier Na^+^/K^+^ balance than untreated ones, implying activation of similar Na^+^ exclusion and K^+^ retention mechanisms. In line with this, a recent study on wheat found that exogenous GABA upregulated the expression of SOS1 and NHX1 in certain salt-stressed cultivars (Badr et al., 2024). By promoting these ion transport pathways, GABA helps to limit the toxic buildup of Na^+^ in the cytoplasm while conserving K^+^, which is vital for enzyme activation and osmotic balance in cells (Chen et al., 2024).

Moreover, GABA’s positive impact on nutrient uptake under salinity extended to micronutrients in our study. Salt stress typically reduces the availability and transport of micronutrients such as Fe, Zn, Mn, and Cu (due to altered soil chemistry and competition from Na^+^/Cl^−^), and we indeed saw 30–40% drops in these elements in untreated plants at high salinity. GABA-treated plants, however, showed significantly higher Fe, Zn, Mn, and Cu concentrations in their tissues compared to non-treated counterparts under the same salt conditions. This suggests GABA helps maintain root functionality and perhaps rhizosphere conditions so that micronutrient uptake continues despite salinity. While specific studies on GABA and micronutrient uptake are limited, the comprehensive analysis by Qian et al. (2024) on soybean did report that GABA application raised the levels of Zn^2+^ and Fe^2+^ in salt-stressed plants, along with other minerals (Qian et al., 2024). They attributed this to GABA’s role in preserving membrane integrity and transporter activities under stress, which would similarly explain our observations. It is conceivable that GABA might modulate the expression or activity of certain micronutrient transporters indirectly through its stress signaling functions, although more research is needed in this area. In summary, our data underscore that GABA fosters ionic equilibrium by lowering Na^+^ toxicity and bolstering the uptake of essential macro- and micro-nutrients under saline conditions. This nutritional homeostasis is a crucial factor that enables better growth and physiological performance in GABA-treated plants facing salt stress.

The various physiological and biochemical improvements observed with GABA treatment from growth and water status to ion balance and oxidative stress mitigation point to a highly integrative mode of action. Rather than affecting a single pathway, GABA appears to orchestrate multiple stress response mechanisms in concert, ultimately leading to enhanced salt tolerance. Our findings can be synthesized into a model where exogenous GABA confers protection through: (1) osmotic adjustment (via increased osmolyte accumulation and improved water retention), (2) ionic homeostasis (via reduced Na^+^ uptake/transport and improved K^+^, Ca^2+^ nutrition), and (3) oxidative stress reduction (via upregulation of antioxidant defenses, both enzymatic and non-enzymatic). These mechanisms are interrelated and together sustain cellular functioning under salt stress. For example, by limiting Na^+^ accumulation, GABA indirectly prevents some ROS generation that would occur from ion toxicity; by maintaining higher K^+^ and Ca^2+^, it helps preserve enzyme activities and membrane stability, which in turn supports better photosynthesis and growth under stress (Zarbakhsh et al., 2025). GABA-driven osmolyte production not only aids in osmotic balance but also directly protects proteins and membranes and can enhance ROS scavenging (Hayat et al., 2023). Meanwhile, the robust antioxidant system induced by GABA shields the membranes and photosynthetic apparatus from oxidative burst damage, ensuring higher membrane integrity (MSI) and chlorophyll retention, thereby contributing to sustained growth and productivity in saline conditions (Badr et al., 2024).

Our results corroborate the emerging consensus that GABA functions as a pivotal stress ameliorating signal in plants. Exogenous GABA has been shown to interact with plant stress hormone signaling and Ca^2+^-dependent signaling networks, thereby modulating downstream stress-responsive gene expression (Kabała and Janicka, 2024). For instance, GABA treatments in wheat were linked to the upregulation of stress-responsive genes (including those for ion transporters and dehydrins) and the downregulation of growth-inhibitory genes, effectively reprogramming the plant’s response to stress in a protective manner (Zarbakhsh et al., 2025). It is also notable that GABA can rapidly accumulate in plants under stress as an endogenous metabolite; thus, applying GABA exogenously likely amplifies a natural defense metabolite signal, kick-starting the plant’s acclimation processes (Guo et al., 2023).

Recent studies have highlighted the potential of GABA as a powerful mitigator of combined salinity and drought stress, owing to its multifaceted roles in osmotic adjustment, redox homeostasis, and nutrient uptake. GABA application has been shown to enhance proline accumulation and antioxidant activity, thereby alleviating oxidative damage and improving physiological resilience in a variety of crops (Qian et al., 2024). Under simultaneous drought and salt stress, which synergistically impair water balance and ion transport, GABA fosters membrane stability, regulates Na^+^/K^+^ homeostasis, and sustains chlorophyll content and photosynthetic efficiency (Dabravolski and Isayenkov, 2023). These findings align with broader literature indicating that moderate GABA concentrations (1–2 mM) are optimal for enhancing stress tolerance across species by modulating metabolic and physiological pathways (Ullah et al., 2023; Hayat et al., 2023).

In addition to its osmoprotective functions, GABA is now recognized as a key signaling molecule in plants, particularly under abiotic stress conditions. It acts upstream of critical secondary messengers such as Ca^2+^, nitric oxide (NO), and ROS, forming part of a complex signaling network that triggers downstream defense responses (Kabała and Janicka, 2024; Guo et al., 2023). GABA-induced signaling has been associated with activation of calcium channels and modulation of the H^+^-ATPase pump, which contributes to ion homeostasis and stress acclimation (Su et al., 2019). Moreover, GABA influences gene expression related to stress tolerance by interacting with redox-sensitive transcription factors and regulating the antioxidant defense machinery (Chen et al., 2024; Dabravolski and Isayenkov, 2023). For example, elevated endogenous GABA levels under stress enhance the expression of genes encoding SOD, APX, and GR, leading to improved detoxification of ROS (Seifikalhor et al., 2019). This signaling role positions GABA not merely as a metabolic buffer but as a central regulator of adaptive plant responses to complex environmental stresses (Shomali et al., 2021).

## 5 Conclusion

Our findings illustrate that γ-aminobutyric acid (GABA) confers multifaceted protection against salinity stress in plants. By enhancing water retention, reinforcing antioxidant defense systems, and modulating ion homeostasis, GABA-treated plants maintained superior growth and physiological performance under saline conditions compared to untreated controls. These results support the role of exogenous GABA as an effective stress-mitigating agent in crop management, particularly in regions affected by soil salinization. The application of GABA represents a practical and eco-friendly strategy to enhance plant resilience under abiotic stress. Future investigations should focus on elucidating the underlying molecular mechanisms by which GABA orchestrates these protective effects especially its influence on hormone signaling pathways and stress-responsive gene networks. Overall, the integrative action of GABA in alleviating osmotic, ionic, and oxidative stress underscores its potential as a powerful tool in developing salt-tolerant agricultural systems.

## Data Availability

The raw data supporting the conclusions of this article will be made available by the authors, without undue reservation.
